# 

**Published:** 1850-04

**Authors:** 


### NOTICE TO CORRESPONDENTS.

Communications and Books for notice should be addressed to the Editor, care of Messrs. Lindsay & Blakiston.

Letters, &c., connected with the *business affairs* of the Journal should be addressed to the Publishers.

Papers for publication must be received *before* the 20th of the month, or they cannot appear in the forthcoming number.

Several of our domestic exchanges have either come very late to hand, or have entirely failed to reach us. Those only that have been received since March 1st are included in the following list.

The Boston Medical and Surgical Journal. (Weekly, Boston.)
Buffalo Journal.
Charleston Medical Journal and Review.
New York Journal of Medicine.
St. Louis Probe.
Northern Lancet.
Medical News.
Western Lancet.
The British American Journal of Medical and Physical Science. (Montreal.)
The London Lancet. (Weekly, London.)
The Medical Times. (Weekly, London.)
London Journal of Medicine, for February and March.
Dublin Medical Press.
Provincial Medical and Surgical Journal.
Dublin Journal.

—

The following works have also been received for notice:

Bell on Baths and Watery Regimen. (From Messrs. Barrington & Haswell.)

Maclise's Surgical Anatomy. Part 2. (From Messrs. Lea & Blanchard.)

Churchill on Diseases of Females. Fifth edition. (From Messrs. Lea & Blanchard.)

Encyclopædia of Chemistry by Booth. (From Mr. H. C. Baird.)

American Medical Formulary. (From Messrs. Lindsay & Blakiston.)

Accommodation of the eye to distance. By W. C. Wallace, M. D. From the Author.

A Memoir on Stricture of the Urethra. By J. P. Mettauer, M. D., LL. D. From the Author.

Address to the Graduates of Pennsylvania Medical College. By Prof. Wiltbank. From the Author.

Medical Reform. By O. W. Taylor, M. D. From the Author.

Lecture introductory to the course of Surgery in Massachusetts Medical College. By H. J. Bigelow, M. D. From the Author.

Observations on planetary and celestial influences in the production of epidemics. By J. S. Bowran, M. D.

Catalogue of the University of Pennsylvania.

Transactions of the Medical Association of Southern Central New York.

The Obstetric Institute of Philadelphia.

Circular of the Medical Institution of Geneva College.

Seventh Annual Report of State Lunatic Asylum, New York.

—

The foreign correspondents of the Examiner will please direct their Exchanges and other communications to the care of Mr. Charles J. Skeet, 27 King William St., Charing Cross, Lond[illegible] or Mr. H. B[illegible]ge, 21 Bis, Quai V[illegible]

### GRADUATES OF JEFFERSON MEDICAL COLLEGE OF PHILADELPHIA.—March, 1850.

At a Public Commencement, held on the 9th of March, 1850, the degree of Doctor of Medicine was conferred on the following gentlemen by the Rev. C. C. Cuyler, President of the Institution; after which a charge to the Graduates was delivered by Professor Mitchell.

| NAME. | STATE. | SUBJECT OF THESIS. |
|---|---|---|
| Aikins, William T. | Canada. | Osteology. |
| Ake, Joseph H. | Pennsylvania. | Puerperal Convulsions. |
| Alcorn, James P. | Pennsylvania. | Lithotomy. |
| Alexander, Richard H. | Kentucky. | Hæmatosis. |
| Anderson, Zebulon M. P. | Virginia. | Cinchona. |
| Ashe, Edmund F. | North Carolina. | Typhoid Fever. |
| Ashe, Richard D. | Alabama. | Congestive Fever. |
| Ashley, Cornelius, | Georgia. | Physiology of the Urine. |
| Austin, Henry O. | Virginia. | Acute Peritonitis. |
| Austin, Peter, | Missouri. | Causæ Morbi. |
| Bache, Thomas Hewson, | Pennsylvania. | Bromide of Potassium. |
| Ballou, Isaac T. | Virginia. | Signs of Pregnancy. |
| Banks, James Oliver, | Alabama. | Pseudo-membranous Laryngitis. |
| Barksdale, Nathaniel, | Virginia. | Fever. |
| Barnes, Laken D. | Kentucky. | Typhoid Fever. |
| Burnley, Hardin, | Mississippi. | Constipation. |
| Barr, Edwin W. | Illinois. | Calorification. |
| Bloodworth, Wiley W. | Georgia. | Typhoid Fever. |
| Boorse, Isaiah H. G. | Pennsylvania. | Medical Skepticism. |
| Bott, James P. | Virginia. | Amenorrhœa. |
| Bowcock, James M. | Virginia. | Concussion of the Brain. |
| Bowland, Milton J. | Ohio. | Unity of Fevers. |
| Brackenridge, Henry H. | Pennsylvania. | Diagnosis of Typhoid Fever. |
| Bradford, Thomas A. | Florida. | Bloodletting. |
| Briggs, George W. (M. D.) | Virginia. | Pneumonia. |
| Bronson, William S. | New York. | The Pulse in Disease. |
| Brooks, William A. | Mississippi. | Remittent Fever. |
| Brown, Neill D. | Mississippi. | Pneumonia. |
| Brown, Robert S. | Pennsylvania. | Puerperal Convulsions. |
| Brown, Solomon, | Connecticut. | Metrorrhagia. |
| Brown, Spencer W. | Missouri. | Retroversio Uteri. |
| Bryant, Cassander E. | Ohio. | Pericarditis. |
| Buckingham, E. Milton, | Ohio. | Hæmatology. |
| Buffington, Thomas C. | Virginia. | Progress of Medicine. |
| Buford, James S. | Mississippi. | Congestive Fever. |
| Bullock, J. Row, | New York. | Dysentery. |
| Burton, Daniel L. | Virginia. | Intermittent Fever. |
| Cake, William M. | Ohio. | Functions of the Nervous System of Organic Life. |
| Carithers, Eli K. | Illinois. | Scarlatina. |
| Chamblin, Marquis R. | Kentucky. | Acute Pleuritis. |
| Chandler, S. Temple, | Virginia. | Influence of Oxygen on the Economy. |
| Chase, Charles T. | New Hampshire. | Empyema. |
| Chester, Samuel H. | Tennessee. | Typhoid Fever. |
| Chorpenning, Frank, | Pennsylvania. | Hygienic Management of Children. |
| Claiborne, J. Herbert, (M.D.) | Virginia. | Uric Acid Diathesis. |
| Clark, Henry, | New York. | Hip Disease. |
| Clark, William L. | Pennsylvania. | Pathology and Treatment of Traumatic Tetanus. |
| Cochran, William S. | Pennsylvania. | Epidemic Cholera. |
| Cockerille, Americus, | Virginia. | Acute Pleuritis. |
| Coombs, David H. | Indiana. | Scarlatina. |

| | | |
|---|---|---|
| Coover, Eli H. | Pennsylvania. | Acute Gastritis. |
| Culbertson, Howard, | Ohio. | Cell Life. |
| Cuthbertson, David H. | North Carolina. | Pathology of Inflammation. |
| Daly, Lafayette, | Virginia. | Acute Bronchitis. |
| Dameron, Robert C. | Mississippi. | Influence of the Mind on the Body. |
| Dana, Marcus, | Ohio. | Endocardial Coagula. |
| Daniel, Milton J. | Georgia. | Natural Labor. |
| Davidson, Junius, | Mississippi. | Opium. |
| Davis, Andrew J. | Pennsylvania. | Vis Medicatrix Naturæ. |
| Derrickson, John B. | New Jersey. | Character of the Medical Practitioner. |
| Dickson, James T. | Missouri. | Meningitis. |
| Dold, Samuel M. | Virginia. | Aneurism. |
| Dorset, John Lewis, | Virginia. | Γνῶθι Σεαυτον |
| Dorsey, John P. | Pennsylvania. | Delirium Tremens. |
| Dossey, George W. | Virginia. | Rubeola. |
| Drummond, John T. | Virginia. | Ileitis. |
| Dunlap, Theodore, | Kentucky. | Pneumonia. |
| Dupree, Ira E. Jr. | Georgia. | Dyspepsia. |
| Durst, Daniel P. | Pennsylvania. | Medicines. |
| Du Val, Lucian Spence, | Florida. | Malaria. |
| Edwards, Americus A. | Missouri. | Indigestion. |
| Elliott, Thomas Balch, | New York. | The Age—its *Pathies* and *Nostrums*. |
| Emmet, Thomas Addis, | Virginia. | The Organic Circle of Nutrition. |
| Ewing, George, | Pennsylvania. | Bloodletting. |
| Fitts, John H. | Alabama. | Reparation of Wounds. |
| Fuqua, Thomas B. | Virginia. | Typhoid Fever. |
| Gale, William H. | Maryland. | Uterine Hemorrhage. |
| Garden, John B. | Virginia. | Pneumonia. |
| Gay, Neil B. Jr. | Virginia. | Dysentery. |
| Gilbert, Michael M. | Kentucky. | Intermittent Fever. |
| Gillam, Louis M. | North Carolina. | Angina Maligna. |
| Gillespie, William A. | Georgia. | Intermittent Fever. |
| Gilmore, William J. | Pennsylvania. | Typhoid Fever. |
| Graham, Edward L. | Virginia. | Placenta Prævia. |
| Graham, Frederick R. | New Jersey. | Epilepsy. |
| Grammer, Jones M. | Virginia. | Aneurism. |
| Grant, Gough W. | North Carolina. | Constipation. |
| Gross, James D. | Pennsylvania. | Peculiarities of the Female Economy. |
| Haile, William J. | Virginia. | Cholera Asphyxia. |
| Hancock, William G. | Virginia. | Cholera. |
| Hann, John A. | Pennsylvania. | Intermittents of Wyoming Valley |
| Hardman, William B. J. | Georgia. | Croup. |
| Hayes, Isham H. | Alabama. | Typhoid Fever. |
| Hendren, Samuel R. | Virginia. | Uterine Hemorrhage. |
| Henry, T. Charlton, | Pennsylvania. | Influence of Atmospheric Temperature on Disease. |
| Heston, Abiah P. | Pennsylvania. | Dysenteric Diarrhœa. |
| Hewson, Addinell, | Pennsylvania. | Prostate Gland. |
| Hirst, Cyrus J. | Pennsylvania. | Influence of the Mind in a Therapeutical Point of View. |
| Holt, Cicero, | Georgia. | Blennorrhœa. |
| Hoyt, Moses C. | New Hampshire. | Opium. |
| Hudson, Robert B. | Virginia. | Intermittent Fever. |
| Hughes, Thomas I. | Virginia. | Pneumonia. |
| Hyde, John H. | Virginia. | Iodine. |
| Jennings, Alvan J. E. | Virginia. | Duties of a Medical Student. |

| | | |
|---|---|---|
| Jones, John T. (M. D.) | Virginia. | A Case resulting in Pneumothorax. |
| Kelly, Charles B. P. | Pennsylvania. | Clothing. |
| Kimmel, Edmund M. | Pennsylvania. | Duodenal Dyspepsia. |
| Kindrick, Cyrus, Jr. | Maine. | Acute Cystitis. |
| King, James E. | North Carolina. | Terebinthina. |
| Kreitzer, Michael C. | Pennsylvania. | Cutaneo-pulmonic Sympathy. |
| Kyle, George P. | Virginia. | Cholera Infantum. |
| Lamar, Thompson B. | Georgia. | Remittent Fever. |
| Lamme, William H. | Ohio. | Indigestion. |
| Leftwich, John W. | Tennessee. | The Liver. |
| Leigh, John Randolph, | Virginia. | Oxygen Gas. |
| Lewis, James M. | Massachusetts. | Crural Hernia. |
| Lewis, Richard E. | Virginia. | Dysentery. |
| Lillington, George, | North Carolina. | Cholera Infantum. |
| Lindley, Alfred H. | North Carolina. | Entozoa. |
| Lindsay, Reuben, | Virginia. | Arsenic and its Compounds. |
| Link, William, | Pennsylvania. | First Principles of Medicine. |
| Linn, Theodore A. | New Jersey. | Typhoid Fever. |
| Logan, Matthew D. | Kentucky. | Phthisis Pulmonalis. |
| Lowman, John, | Pennsylvania. | Effects of Calomel on the Blood and Liver. |
| Lungren, Samuel S. | Pennsylvania. | Chemical and Medical properties of Oxygen. |
| M'Conaughy, Robert, | Pennsylvania. | Polyæmia. |
| M'Culloch, William P. | Pennsylvania. | Action of the Heart. |
| M'Donald, Nesbit, | Pennsylvania. | Internal Use of Argenti Nitras. |
| M'Veigh, William H. | Virginia. | Symptomatology. |
| Mackie, John Howell, | Massachusetts. | Tetanus. |
| Madison, James A. | Virginia. | Inflammation. |
| Manahan, Valentine, | New Hampshire. | Necrosis. |
| Marks, Julian C. | Virginia. | Phthisis Pulmonalis. |
| Martin, Algernon S. | Virginia. | Origin of the Motor Forces. |
| Meranda, Isaac, | Indiana. | Ergota. |
| Meriwether, John H. | Tennessee. | Acute Pleurisy. |
| Mettauer, Edward M. | Virginia. | Enteric Fever. |
| Michie, John Augustus, | Virginia. | Oxygen Gas. |
| Miller, David H. | Pennsylvania. | Inguinal Hernia. |
| Millner, James S. | Virginia. | Gonorrhœa. |
| Mills, Samuel R. | Virginia. | Intermittent Fever. |
| Mitchell, S. Weir, | Pennsylvania. | The Intestinal Gases. |
| Morehouse, George R. | New Jersey. | Acute Rheumatism. |
| Morrow, William, | Indiana. | Abortion. |
| Moseley, Daniel Willis, | Virginia. | Scarlatina. |
| Mowry, John N. | Pennsylvania. | Pneumonia. |
| Neilson, Robert, | Pennsylvania. | Dysentery. |
| Nightingale, Henry B. | Pennsylvania. | Certain Indigenous and Naturalized Plants of America. |
| Owen, Joseph D. | Georgia. | Insanity. |
| Palmore, Charles R. (M. D.) | Virginia. | Difficulty of Diagnosis of Exanthematous Diseases in the Negro. |
| Parke, Clifford D. | Alabama. | Scrofula. |
| Patton, Joseph C. | Tennessee. | Pneumonia. |
| Peebles, John H. M. | Pennsylvania. | Erysipelas. |
| Piggot, William M. | Virginia. | Rubeola. |
| Powell, H. Brooke, | Virginia. | Oleum Morrhuæ. |
| Prescott, Paul T. | Maine. | Ascites. |
| Price, Jacob, | Pennsylvania. | Use of Anæsthetics in Childbirth. |
| Quarles, Mercer W. | Virginia. | Typhoid Fever. |
| Quick, Lavington, | England. | Phthisis Pulmonalis. |

| | | |
|---|---|---|
| Rankin, Clarke D. | Pennsylvania. | Cod-liver Oil. |
| Rhinehart, Solomon E. | Ohio. | Malaria. |
| Ricketts, Gerard C. | Virginia. | Colo-rectitis. |
| Roberts, Benjamin F. | Connecticut. | Rubeola. |
| Rogers, Thomas H. | Virginia. | Typhoid Fever. |
| Ross, Samuel M. | Pennsylvania. | Typhus Fever. |
| Russell, William M'N. | Mississippi. | Cholera in Claiborne Co. Mississippi, in 1849. |
| Schriver, Albert, | Pennsylvania. | The Kidney and its Secretion. |
| Seller, Theophilus S. | Indiana. | Mercury. |
| Sharp, Alexander E. | Pennsylvania. | Erysipelas. |
| Shelly, Aaron F. | Pennsylvania. | Delirium Tremens. |
| Sherrell, Joseph L. | Tennessee. | Oxygen Gas and its Uses. |
| Shewalter, George William, | Virginia. | Inguinal Hernia. |
| Shoyer, Charles C. | Wisconsin. | Intermittent Fever. |
| Smith, Frisby T. | Pennsylvania. | Hysteria. |
| Smith, George L. | Ohio. | Scarlatina. |
| Smith, John H. | Virginia. | Typhoid Fever. |
| Stewart, William G. | Pennsylvania. | Dysentery. |
| Stuart, Joseph G. | Indiana. | Cholera. |
| Sturdivant, Marcus, | Virginia. | Acute Pleurisy. |
| Taliaferro, Philip A. | Virginia. | Advance of Medicine and Consequent Improvement in the Knowledge of the Nature and Treatment of the Uric Acid Diathesis. |
| Taylor, Thomas W. | Pennsylvania. | Ligation of the Arteries. |
| Temple, Thomas P. | Virginia. | Puberty. |
| Thorndike, Albert. | Maine. | Tetanus. |
| Todd, Louis H. | Kentucky. | Epidemic Cholera. |
| Trammell, Francis A. | Alabama. | Anæmia. |
| Tucker, Thomas W. | Virginia. | Acute Colitis. |
| Tuttle, Levi W. | New Hampshire. | Scarlet Fever. |
| Urquhart, George, | Pennsylvania. | Acute Hydrocephalus. |
| Voorhees, Charles H. | New Jersey. | Spermatorrhœa. |
| Wakefield, Mathew F. | Kentucky. | Purpura Hæmorrhagica. |
| Walker, James R. | Virginia. | Oxygen Gas. |
| Wallton, James C. | Virginia. | Opium. |
| Ward, Edward W. | North Carolina. | Puerperal Fever. |
| Warfield, Milton, | Maryland. | Typhoid Fever. |
| Webber, Joseph B. | Maine. | Strumous Ophthalmia. |
| Weiser, Charles S. | Pennsylvania. | Ascites. |
| Westmoreland, Willis F. | Georgia. | Prolapsus Uteri. |
| Whitaker, John F. | Arkansas. | Intermittent Fever. |
| Wilbur, Greenleaf A. | Maine. | Rubeola. |
| Williams, Lorenzo D. (M.D.) | Virginia. | Gonorrhœa. |
| Wilson, Edward A. | Virginia. | Vis Medicatrix Naturæ. |
| Wilson, John R. | Pennsylvania. | Eclampsia. |
| Woodbury, Jonathan, | Nova Scotia. | Indigestion. |
| Woddrop, Henry, | Pennsylvania. | Nature and General Phenomena of Fevers. |
| Wood, Edwin N. (M. D.) | Virginia. | Pneumonia. |
| Wood, John Dean, | Virginia. | Surgical Modes of Arresting Hemorrhage. |
| Woodside, James A. | South Carolina. | Typhoid Fever. |
| Worl, Eli T. | Pennsylvania. | Quackery. |
| Wright, David D. | Canada West. | A Paroxysm of Intermittent Fever. |
| Young, George W. | Georgia. | Obstructed Labour. |

James W. Lugenbeel, M. D., of Virginia, late physician to the Colony of Liberia, was admitted to the ad eundem degree of Doctor of Medicine. Total, 211.

R. M. HUSTON, M. D., Dean of the Faculty.

